# Supplementary material for: Generating Findings for Jaw Cysts in Dental Panoramic Radiographs Using a GPT-Based VLM: A Preliminary Study on Building a Two-Stage Self-Correction Loop with a Structured Output (SLSO) Framework
Source: Diagnostics (Basel). 2026 Apr 5;16(7):1096. doi: 10.3390/diagnostics16071096 (PMC13073833; doi:10.3390/diagnostics16071096)
Supplement: Supplementary file 1 [file diagnostics-16-01096-s001.zip › Supplementary Information_v4.pdf]

Supplementary Information

Generating Findings for Jaw Cysts in Dental Panoramic Radiographs Using a GPT-Based VLM: A Preliminary Study on Building a Two-Stage Self-Correction Loop with Structured Output (SLSO) Framework

Nanaka Hosokawa[1*], Ryo Takahashi[2], Tomoya Kitano[3], Yukihiro Iida[3], Chisako Muramatsu[4], Tatsuro Hayashi[2], Yuta Seino[5], Xiangrong Zhou[6], Takeshi Hara[6], Akitoshi Katsumata[3], and Hiroshi Fujita[6*]

1 Graduate School of Natural Science and Technology, Gifu University, Gifu, Japan
2 EyeTech Co., Ltd, Tokyo, Japan
3 School of Dentistry, Asahi University, Mizuho, Japan
4 Faculty of Data Science, Shiga University, Hikone, Japan
5 Division for Oral Dental Informatics, The University of Osaka Dental Hospital, Osaka, Japan
6 Faculty of Engineering, Gifu University, Gifu, Japan
* Correspondence: hosokawa.nanaka.u9@s.gifu-u.ac.jp (N.H.); hiroshi.fujita.gifu@gmail.com (H.F.)

Supplementary Note 1 - Preliminary Experiments

This note describes the preliminary phase of the study design, presents exploratory experiments that motivated the development of the proposed method, and summarizes their outcomes.

Pilot Experiments

To develop a dental-image diagnosis-support system using GPT-4o, we incrementally improved the input image data preprocessing stage and prompt design, clarifying the issues at each stage before arriving at the final method design. The following section describes the considerations of each of the four phases.

Phase 1: Direct Findings Generation Using Entire Image

In the pilot experiments, we compared the use of entire dental panoramic radiographs

[**Figure S1. (a)**; original image size: 1976 × 976 pixels] sized to 224 × 224, 512 × 512, and 1024 × 1024 pixels. For image resizing, we zero-padded above and below the shortest edge (976 pixels vertically) of the original image to create a 1976×1976-pixel square image and then used bilinear interpolation to shrink the image to the target size at the same ratio in both width and height. This method minimizes the distortion of anatomical structures while maintaining aspect ratio.

For each image size, we evaluated GPT-4o's finding generation using a standard prompt: "Please write a radiological finding description for the cyst shown in this dental panoramic radiograph, including its location, boundary, internal structure, and impact on the surrounding area."

As a result, we concluded that a 512×512-pixel image was the optimal setting, which had the lowest rate of error responses such as "I'm not a doctor, so I can't answer." We then directly input a 512 × 512-pixel image into GPT-4o to generate a cyst finding.

Specific example results:

Ground truth: "A clearly demarcated, unilocular radiolucency is observed from teeth 45 to 47."

GPT-4o output: "There appears to be a radiolucent lesion in the left mandibular molar region, with a somewhat indistinct border, and possible effects on the adjacent teeth."

The results of Phase 1 identified the following issues:

1) Processing load issues due to large image size.

2) Due to the diverse anatomical structures contained throughout the image, GPT-4o was distracted by findings other than cysts (crown restorations, implants, etc.).

3) Tooth number could not be identified (ambiguous expressions such as "left mandibular molar area").

4) Frequent use of vague expressions that are difficult to evaluate, such as "somewhat," "seems to be," and "possibly".

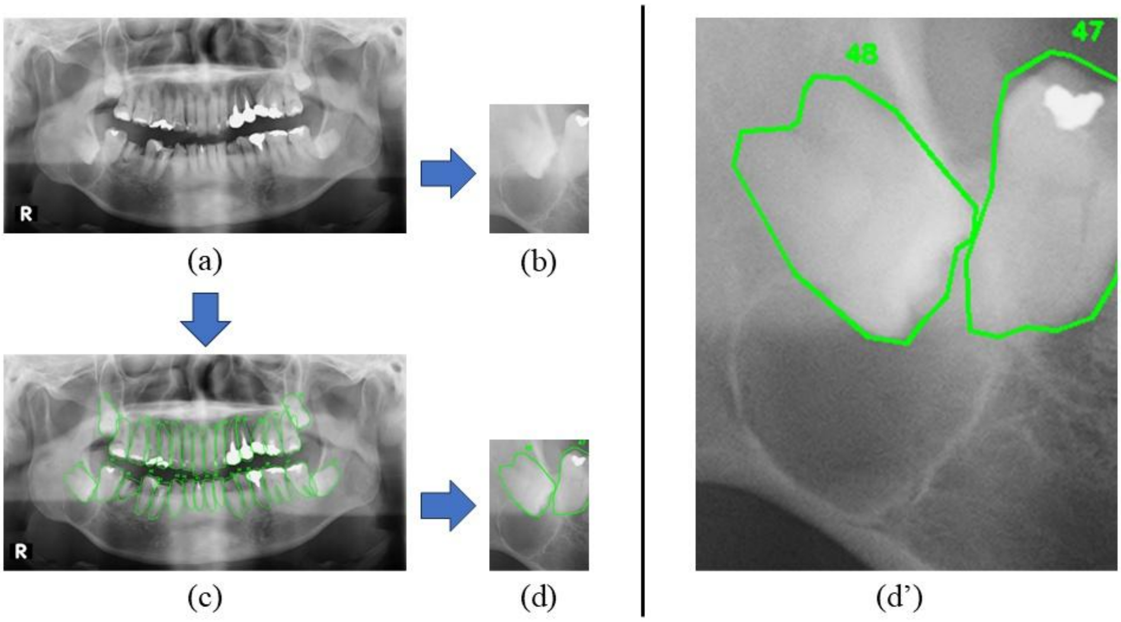

Figure S1. Extraction of cyst regions from dental panoramic radiographs.

The preprocessing of panoramic radiographs is shown. (a) Original image (1976×976 pixels). (b) Simply cropped ROI image around the cyst (200-400 pixels, preserving the original resolution). (c) The original image in (a) with annotations (green outlines and FDI tooth numbers) of all tooth margins. (d) The cropped ROI image shown in (b), with annotated tooth margins and tooth numbers. (d') Enlarged view to make the tooth numbers around the cyst more visible.

Phase 2: Trial Improvements through Manual Extraction of ROIs

In response to the challenges faced in Phase 1, we considered a method in which dentists manually extracted regions of interest (ROI) around the cysts [approximately 200–400 pixels; **Figure S1. (b)**] and fed the target area into the GPT-4o. While maintaining the basic structure of Phase 1, the verbal prompt was modified to reflect the characteristics of the ROI image: "Please describe in detail the location, boundaries, internal structure, and impact on surrounding tissues of the cyst in this extracted dental image region," encouraging precise observation within a limited area. While ROI extraction improved processing speed and significantly reduced the error rate compared to full image input, there were still many errors regarding cyst location and number of affected teeth (FDI number).

This manual ROI extraction process can be automated by incorporating a conventional deep learning-based cyst detection method. In recent years, research on the automated detection of cysts in dental panoramic radiographs has actively progressed.

Examples include an automated jaw cyst detection and classification system using a CNN architecture with an improved YOLOv3 architecture [16], a real-time cyst detection method using YOLOv2[17], and a segmentation method for odontogenic cyst lesions using U-Net-based multistage adaptive network[18]. An automated system for detecting the nasopalatine duct and apical cysts using EfficientDet-D3 achieved an mAP of 93.8%[19]. By integrating these technologies, the structured data generation framework proposed in this study is expected to be fully automated.

Specific example results:

Ground truth: "Clearly defined translucent regions are observed around no. 46 and no. 47."

GPT-4o output: "A radiolucent lesion is identified in the region of tooth #46, with a similar appearance observed adjacent to tooth #47."

The results of Phase 2 identified the following issues:

1) Although the accuracy of tooth number identification improved, ambiguous location expressions such as "vicinity" and "nearby" still remained.

2) It is difficult to grasp the exact positional relationship with adjacent teeth.

Phase 3: Incorporation of Tooth Segmentation and Tooth Number Annotation

To address the issue of positional relationship recognition in Phase 2, we considered an input format that combined the ROI images and affected tooth number information. Ideally, tooth segmentation and automatic FDI number assignment would be achieved using conventional deep learning techniques[20]. However, in this study, as proof-of-concept, we verified the effectiveness of the proposed method using manual annotation by a dentist. **Figure S1. (c)** shows the original image annotated with tooth margins and tooth number, while **Figure S1. (d)** and **(d')** show the cropped ROI highlighting the cyst and affected teeth, with (d') providing an enlarged view for clarity. This representation is expected to provide the cyst and affected tooth information in a format that can be visually interpreted by the GPT model.

Specific example results:

Ground truth: "Clearly demarcated, unilocular translucencies are observed around teeth #46 and #47."

GPT-4o output: "A radiolucent area is observed around teeth #46 and #47, with relatively well-defined borders and showing mild to moderate

radiolucency."

The results of Phase 3 identified the following issues:
1) The accuracy of tooth number identification improved (correctly identifying "teeth #46 and #47").
2) Frequent use of ambiguous terms such as "relatively" and "mild to moderate" in descriptions of radiological features.
3) Occurrence of hallucination (unspecified content).

Phase 4: Introducing CoT Prompts

To mitigate the hallucination problem identified in Phase 3, we introduced CoT prompts that clearly explained the thought process step-by-step and explored methods to encourage stepwise reasoning in GPT-4o. Specifically, each item in the schema defined in **Table 1.** (radiographic transparency, internal structure, boundary, root resorption, tooth displacement, relationship to the mandibular canal and maxillary sinus, and affected tooth number, see Section 2.3 of the main text) was explicitly presented as an option, and the GPT-4o was instructed to simultaneously select an option for each item and write a final statement of findings within a single prompt.

Example of a specific CoT prompt:

"Analyze the cyst in this dental panoramic radiograph step by step using the following steps:
Step 1: Determine radiolucency (radiolucent/radiopaque).
Step 2: Evaluate the internal structure (unilocular/multilocular).
Step 3: Evaluate the clarity of the borders (well-defined/ill-defined).
Step 4: Evaluate the presence and extent of root resorption (no/mild/severe).
Step 5: Evaluate the presence and extent of tooth displacement (no/mild/severe).
Step 6: Evaluate the relationship with the mandibular canal and maxillary sinus (contact/displacement/invasion).
Step 7: Identify the affected tooth number (listed using FDI numbers).
Finally: Create a radiological statement based on the results of the above analysis."

Similar to Phase 3, the ROI images were provided with FDI tooth number annotations and margin information for each tooth. While this improved the transparency of the reasoning process, it did not fundamentally resolve the issues of ensuring the factuality of the final findings or the verifiability of the written content. The results of the

comparison between this CoT method and the structured method described below are detailed in Section 3.

Specific example results:

Ground truth: "A clearly demarcated unilocular radiolucency is observed around teeth numbers 46 and 47."

GPT-4o output (CoT): "First, the location is assessed: a radiolucent area is observed in the mandibular molar region. Next, the border is evaluated: the margin appears to be well-defined, although not with certainty. The radiolucency is then judged: it seems to be of moderate degree. In conclusion, there is a possible cyst-like lesion around tooth #46."

The results of Phase 4 identified the following issues:

1) The transparency of the thought process was improved by stepwise reasoning.
2) Frequent use of uncertain expressions such as "seems," "appears," and "possibly."
3) It was difficult to guarantee the factuality of the final findings.
4) The issue of the verifiability of the written content was not fundamentally resolved.

Supplementary Note 2 - Detailed Description of Proposed Framework

This supplementary note provides a detailed description of each step (1–10) of the proposed framework, including the representative prompts, schema definitions, and example outputs. These details complement the overview presented in the main text (Section 2.4, **Figure 1.**).

Step 1: Input

ROI images annotated with tooth margins and FDI numbers surrounding the jaw cyst are input along with verbal prompts containing interpretation instructions.

Example prompt:

"You are a professional dental radiologist. Analyze this dental panoramic radiograph and output the results as structured data according to the specified items."

Step 2: GPT-4o Image Analysis (Start of Parallel Processing)

Utilizing the GPT-4o multimodal capabilities, the ROI image is analyzed. From this step onward, parallel processing is employed while simultaneously performing structured data generation (Step 3) and tooth number extraction (Step 4).

Step 3: Structured Data Generation

GPT-4o is instructed to generate structured data (in JSON format) according to the schema defined in **Table 1**. Specifically, the interpretation item schema was defined using the Pydantic library, and the radiological features of the lesion (jaw cyst) in the image were extracted as strictly structured data by setting the Pydantic schema in OpenAI API (Application Programming Interface), response format parameters.

Pydantic schema definition example:

```
class CystAnalysis(BaseModel):
    radiolucency: Literal["radiolucent", "radiopaque"]
    internal_structure: Literal["unilocular", "multilocular"]
    boundary: Literal["well-defined", "ill-defined"]
    root_resorption: Literal["no", "mild", "severe"]
    # ...
```

Example output:

```
{
 "radiolucency": "radiolucent",
 "internal_structure": "unilocular",
 "boundary": "well-defined",
 "root_resorption": "mild",
 "tooth_displacement": "no",
 "anatomical_relation": "contact",
 "affected_teeth": ["33", "34", "35", "36"]
}
```

Step 4: Tooth Number Extraction

In parallel, GPT-4o's visual recognition capabilities are used to extract textual tooth numbers from the input image for teeth suspected to be affected by jaw cysts. This information is obtained separately from the structured data and used for the subsequent consistency assessment.

Step 5: Tooth Number Consistency Check

The inclusion and consistency of the number of affected teeth described in the structured data obtained in Step 3 and the tooth number list extracted in Step 4 are evaluated. For example, if the structured data describes "33, 34" and the extracted results from the image are "33, 34, 35, 36," this is considered a match. However, if a complete mismatch exists, regeneration is required.

Step 6: Regenerate Structured Data & Re-extract Tooth Numbers

If an inconsistency is recognized in Step 5, GPT-4o is instructed to regenerate the structured data, as shown in the following example. In this case, specific feedback information regarding tooth number mismatch is created, including 1) the tooth number identified in the structured data, 2) the tooth number extracted directly from the image, and 3) a prompt clearly indicating the differences between the two, and the structured data is regenerated. Subsequently, the system returns to Step 5 and repeats the consistency check.

Example prompt:

"An inconsistency in tooth numbers was detected in the previous structured data

generation.

Tooth numbers identified in the structured data: [31, 32]

Tooth numbers extracted directly from the image: [11, 12, 13, 21, 22, 23]

Difference: Numbers 11, 12, 13, 21, 22, and 23 are not included in the structured data. Please recheck the image and regenerate structured data in JSON format, including all teeth affected by the lesion."

Step 7: Finding Generation

Based on the structured data, whose consistency was confirmed, GPT-4o is instructed to generate findings in a natural language format. In this case, the generated findings mimicked the actual radiographic findings written by dentists, aiming for quality that can be used directly in clinical reports.

Example prompt:

"Based on the following structured data, please output a dental radiology finding sentence in natural-sounding Japanese medical description. Please standardize location expressions in the format of 'from number XX to number XX' and create a concise, clinically appropriate finding sentence that includes transparency, boundary, and internal structure. [Insert JSON of tooth number consistency-checked structured data]"

Step 8: Regenerate Structured Data from Findings

The sentence generated in Step 7 is re-input into GPT-4o, and the information contained in the sentence is converted back into structured data. This enables the system to verify whether the natural language representation accurately reflects the original structured information.

Example prompt:

"Parse the following dental radiological findings and extract the information contained therein as structured data (JSON format). Output the following items according to the defined schema: radiolucency, internal structure, boundary, root resorption, tooth displacement, anatomical relationship, and affected tooth number. [Insert the findings generated in Step 7]"

Step 9: Structured Data Consistency Check

The consistency between the data restructured in Step 8 and those generated in Step

3 is verified for the following items:

- Matching of affected tooth numbers: Detection of missing tooth numbers.
- Match radiological findings such as position, translucency, internal structure (unilocular/multilocular), and boundary.
- Semantic discrepancies: identification of inconsistencies arising from ambiguous terms or synonymous substitutions.

If consistency is confirmed, the findings are produced as the final output.

Step 10: Regeneration Findings

If an inconsistency was detected in Step 9, GPT-4o was used to regenerate the findings. Structured data (consistency verified in Step 5 and corrected in Step 6 if necessary) are retained, while only the findings are regenerated, with feedback specifying the inconsistency (e.g., "The tooth number is missing" or "The boundary description differs from the structured data"). The regenerated findings are then reverified for consistency in Steps 8 and 9.

Example prompt:

"An inconsistency issue was detected in the previously generated findings. Specifically, it is [content of the inconsistency]. Please regenerate the findings accurately based on the following structured data:
[Insert tooth number consistency-checked structured data]"
